# Varicella in Adults

**Published:** 1901-05-25

**Authors:** 


### VARICELLA IN ADULTS.

According to Dr. Doty, health officer to the Port of New York, a widespread belief exists that varicella does not affect adults, but is confined to infancy and childhood. This, however, is not true, and it is fully understood by those familiar with these diseases that chicken-pox may occur after puberty. The diagnosis between small-pox and chicken-pox depends, he says, upon (1) the character of the eruption, (2) the manner in which it appears, and (3) its distribution or location. In regard to the manner in which it appears in small-pox, the eruption presents itself in one crop, and passes through its different stages practically together, as papules, vesicles, and pustules. In some parts of the body the formation of each stage, or the transition from one stage to the other, may be a little more rapid than others; but when this is finished the eruption is complete, and secondary crops never appear. In chicken-pox, on the other hand, the eruption comes out in a manner diametrically the opposite of this. Alongside of vesicles which have dried down with the formation of a black or dark scab we find small tender vesicles just appearing, which are torn by a slight touch of the nail. Moreover the eruption of chicken-pox appears abruptly as vesicles, and is not preceded by papules as in small-pox. As to the distribution of the eruption, in small-pox, even in mild cases, the hands and feet are to some extent almost always involved; whereas in chicken-pox, even with a profuse eruption, the hands and feet are either unaffected or have but little eruption. The appearance of hard, tough, circumscribed, and distended papules or vesicles on the hands or feet, particularly on the palms and soles, is an exceedingly important diagnostic sign of small-pox. Even in mild cases we are quite sure to find a few along the fingers and toes and palms and soles, while in varicella the hands and feet are singularly free, even when the eruption is profuse on other parts of the body. It may be added that the back presents the best surface on which to study the eruption of varicella. As to umbilication this is a well marked and valuable diagnostic sign in small-pox, but what will pass for and is often regarded as umbilication is frequently found in chicken-pox as well as in some forms of syphilitic eruption. Under all circumstances it should be borne in mind that the diagnosis of small-pox and chicken-pox is made from the appearance of the eruption. The constitutional symptoms are important as corroborative evidence, but they should be considered after the eruption has been studied. The more proficient one becomes in the diagnosis of small-pox or chicken-pox the less he relies on constitutional symptoms.

